# Effectiveness of Ezetimibe in Reducing the Estimated Risk for Fatal Cardiovascular Events in Hypercholesterolaemic Patients with Inadequate Lipid Control While on Statin Monotherapy as Measured by the SCORE Model

**DOI:** 10.4061/2011/597163

**Published:** 2010-10-31

**Authors:** John S. Sampalis, Stéphane Bissonnette, Stella Boukas

**Affiliations:** ^1^Departments of Surgery and Epidemiology & Biostatistics, McGill University, Montreal, QC, Canada; ^2^JSS Medical Research Inc., 4492 St., Catherine Street West, Westmount, QC, Canada H3Z 1R7; ^3^Merck & Co, 16711 Trans Canada Highway, Kirkland, QC, Canada

## Abstract

*Objectives*. The aim of this prospective cohort, multicentre study was to assess the effect of coadministrating ezetimibe 10 mg/day with an ongoing statin on the estimated risk for Cardiovascular (CVD) mortality in patients with persistently elevated LDL-C after statin monotherapy. *Methods*. The Systematic Coronary Risk Evaluation (SCORE) function was used to estimate the 10-year risk for cardiovascular mortality at baseline and 6 weeks. Primary outcome measures were absolute and percent changes in estimated Coronary Heart Disease (CHD) Mortality Risk, and general CVD Mortality Risk (Total CVD Mortality Risk). *Results*. 825 patients were included in the analysis. Mean (SD) age was 62 (10.5) years and 62.3% were males. The mean (SD) estimated Total CVD Mortality Risk decreased from 0.068 (0.059) at baseline to 0.053 (0.046) at 6 weeks (RR = 0.77; 95% CI:0.689–0.867), while the estimated CHD Mortality Risk decreased from 0.047 (0.040) at baseline to 0.034 (0.029) at 6 weeks (RR = 0.72; 95% CI:0.624–0.826). *Conclusions*. Co-administration of ezetimibe with a statin is effective in significantly reducing the estimated risk for cardiovascular mortality as measured by the SCORE model.

## 1. Introduction

Cardiovascular disease is the major cause of mortality in Canada, accounting for one-third of all deaths, with an incidence expected to increase within the next decade [1]. Increased serum cholesterol, particularly low-density lipoprotein cholesterol (LDL-C), is directly associated with an increased risk for cardiovascular disease (CVD) [2–5]. Initiation of lipid-lowering pharmacologic intervention for the management of hypercholesterolaemia is generally dependent on the individual patient's estimated risk for cardiovascular events [3, 6, 7]. Ultimately, the aim of lipid-lowering treatment is to effectively reduce the individual patient's risk for CVD, thus decreasing related mortality, morbidity and burden of illness. A cardiovascular risk prediction model was developed by the SCORE (*S*ystemic *CO*ronary *R*isk *E*valuation) project group in accordance to the recommendations from the Second Joint Task Force of European and other Societies on Coronary Prevention [7]. The SCORE model is based on pooled data from 12 European cohort studies, including data on over 205,000 individuals and representing 2.7 million person-years of followup. The model predicts the individual's 10-year risk for fatal cardiovascular events on the basis of age, gender, smoking status, systolic blood pressure (SBP), and total cholesterol (TC). The total SCORE risk is further subdivided into the risk for fatal CHD and other non-CHD cardiovascular death. 

First-line pharmacotherapy for the management of hypercholesterolaemia typically involves lipid-lowering treatment with statins [3, 8, 9]. Despite the effectiveness of statins in reducing serum LDL-C levels, the results of large clinical trials and epidemiological studies have shown that a significant proportion of patients does not achieve target LDL-C levels while on treatment with statins [10–12]. For these patients who remain at increased risk for CVD, combination therapy with additional agents inhibiting cholesterol absorption or bile acid reabsorption, or concomitant use of niacin is recommended by the Canadian Cardiovascular Society [9]. Ezetimibe is a lipid-lowering compound that inhibits cholesterol absorption from the small intestine without affecting the uptake of triglycerides (TG) or fat-soluble vitamins [13]. Co-administration of ezetimibe with statins has been shown to be a potent dual-target strategy, providing enhanced reduction of LDL-C and improvement of the lipid profile over statin monotherapy [14–19], while potentially decreasing the risk of adverse events associated with high-dose statins through moderating the dose of co-administrated statin [20–22]. 

The Ezetrol Add-On study was a multicentre, prospective, Canadian cohort study involving 837 patients who had not achieved target LDL-C levels while on statin monotherapy [23]. The results of this study showed that addition of ezetimibe to the patients' existing statin regimen for six weeks was effective in significantly reducing LDL-C by a mean of 30%, from 3.43 to 2.83 mmol/L. The purpose of the current analysis was to assess the change in predicted risk for fatal CVD as estimated by the SCORE model in the Ezetrol Add-On cohort of patients. 

## 2. Methods

### 2.1. Study Design

The Ezetrol Add-On-Study was a prospective, single-cohort, open-label study conducted in the offices of Canadian general practitioners. Study design details are published elsewhere [23].

In brief, all patients were treated with ezetimibe 10 mg/day co-administered with their existing statin regimen for six weeks. There were no limitations on the type or dose of statin used other than maintaining the same regimen for the duration of the study. The final study assessment took place six weeks (±4 days) after the baseline visit. Patients provided written informed consent prior to participation in the study. The study was approved by two independent ethics review boards (IRB services in Aurora, Ontario and the College of Physicians and Surgeons of Alberta [CPSA]).

### 2.2. Sample Selection

Patients were enrolled by 221 participating physicians. Eligible patients were ≥18 years old with confirmed hypercholesterolaemia defined as LDL-C ≥2.5 mmol/L for patients at high 10-year coronary artery disease (CAD) risk, LDL-C ≥3.5 mmol/L for moderate risk patients and LDL-C ≥4.5 mmol/L for low-risk patients [8]. 

Patients were excluded from the study if they had any condition that would render the patient unable to complete the study or for which study participation would produce significant risk or not be in the best interest of the patient [23]. Concomitant use of any medications that may interact negatively with statins or ezetimibe or affect the patient's serum lipid levels was prohibited.

Patients treated with cardiovascular medications were included in the study provided that they were on a stable medication regimen for at least 4 weeks prior to study entry and remained on the same regimen for the duration of the study.

### 2.3. Outcome Measures

Estimated 10-year risks for fatal cardiovascular event in general CVD (Total CVD Mortality Risk) and for CHD (CHD Mortality Risk) were estimated using the SCORE function. The SCORE risk estimation is based on the individual's age, gender, smoking status, TC, and SBP [7]. The Total CVD-Risk estimate is comprised of the risk for fatal CHD (CHD Mortality Risk) and the risk of fatal non-CHD cardiovascular events. The CHD Mortality Risk and Total CVD Mortality Risk for each subject were estimated at baseline and at six weeks of followup. The primary outcome measures of the analysis were the absolute and percent changes in estimated CHD Mortality Risk and Total CVD Mortality Risk from baseline to the six-week follow-up visit.

### 2.4. Statistical Methods

The intention-to-treat (ITT) principle, including all study patients completing the six-week visit assessment, regardless of compliance with the study protocol, was applied in the analysis. Patients who were lost to followup and did not return for the six-week assessment could not be included in the analysis. The statistical significance of the absolute and percent changes in estimated CHD Mortality Risk and Total CVD Mortality Risk were assessed with the Student's *t*-test for paired samples. The analysis was conducted for the study sample as a whole and stratified by the presence of hypertension, type II diabetes mellitus, the metabolic syndrome and smoking. The metabolic syndrome was defined according to the American Heart Association (AHA) criteria published at the time of the study [24], while hypertension was based on the diagnosis of the treating physician. Current smoking status was ascertained by the treating physician during the screening visit and was assumed to be unchanged during the course of the study. The number of CHD and total CVD deaths per 100,000 at baseline and six weeks was estimated on the basis of the risk estimates. Relative risk (RR) estimates with 95% confidence intervals (CI) were used to assess the precision and statistical significance effect of the change in the estimated CHD and total CVD mortality rates.

## 3. Results

A total of 1,141 patients were screened between November 2003 and April 2005, among which 953 (83.5%) fulfilled the study inclusion criteria and were enrolled in the study. Of the 953 patients enrolled, 825 (86.6%) completed the six-week follow-up with sufficient data for SCORE risk estimation at baseline and at six weeks. There were 128 patients who discontinued from the study prior to the follow-up assessment and cannot be included in this analysis since change in the outcome parameters could not be computed. These included 50 (5.2%) patients who were lost to followup, 45 (4.7%) who were withdrawn by the study investigators because they changed or stopped their statin treatment, 19 (2.0%) who withdrew due to adverse events, 2 (0.2%) who withdrew consent prior to initiation of treatment, and 12 (1.3%) who were missing data for SCORE estimation.

The mean (SD) age of the study sample was 62 (10.5) years with a range between 21 and 89 years, while 62.3% of the patients were male. The most frequently reported comorbidities and risk factors were hypertension (*n* = 423, 51.3%), type II diabetes mellitus (*n* = 342, 41.5%), the metabolic syndrome (*n* = 395, 47.9%), and smoking (*n* = 188, 22.8%). Furthermore, 489 (59.3%) patients had a known family history of CVD and 375 (45.5%) patients of CAD. For the 825 patients included in the analysis, the distribution of statins used during the study period was atorvastatin in 412 (50.5%), followed by simvastatin in 162 (19.6%), rosuvastatin in 118 (14.3%), pravastatin in 102 (12.4%), lovastatin in 24 (2.9%), and fluvastatin in 7 (0.8%) patients. Prior to study enrolment, 328 (39.8%) patients were treated with a moderate or high statin dose, defined as 40 mg/day or 80 mg/day depending on the specific statin. 

The changes in the modifiable risk factor variables applied in the SCORE calculations are shown in Table 1. After six weeks of treatment, a significant mean decrease in SBP from 132.0 to 128.2 mmHg was observed (*P *<  .001) with the largest absolute decrease of −6.59 mmHg (95% CI: −5.25 to −7.94) observed in patients with hypertension. A significant decrease was similarly observed for TC, which decreased from 5.60 to 4.40 mmol/L (*P *<  .001). The largest absolute TC decrease of −1.33 mmol/L (95% CI: −1.47 to −1.19) was observed in these patients who were smokers at the time of the study (Table 1).

At baseline, the mean 10-year estimated CHD Mortality Risk for the study sample was 0.047 (47/1,000 patients), which decreased to 0.034 (34/1,000 patients) after six weeks of treatment (Table 2). Both the mean absolute decrease of −0.013 (95% CI: −0.015 to −0.012), or 13/1,000 patients, and the mean percent decrease of −0.254 (95% CI: −0.271 to −0.238), were statistically significant (*P *<  .001). A similar significant decrease in estimated CHD Mortality Risk was observed for all four patient subgroups analyzed (Table 2). The highest absolute and percent change was observed for smokers with a mean absolute decrease in CHD SCORE of 0.018 and mean percent decrease of −30.3%. 

After six weeks of treatment, a statistically significant decrease in the mean estimated Total CVD Mortality Risk was also observed (*P *<  .001). For the total study sample, the mean estimated Total CVD Mortality Risk decreased from 0.068 (68/1,000 patients) at baseline to 0.053 (53/1,000 patients) after six weeks of treatment (Table 3). Both the mean absolute and percent decrease of −0.015 (15/1,000 patients) (95% CI: −0.017 to −0.013) and −0.198 (95% CI: −0.215 to −0.181), respectively, were statistically significant (*P *<  .001). The highest decrease in estimated Total CVD Mortality Risk was observed for the subgroups of patients with hypertension and smokers.

**Figure 1:**
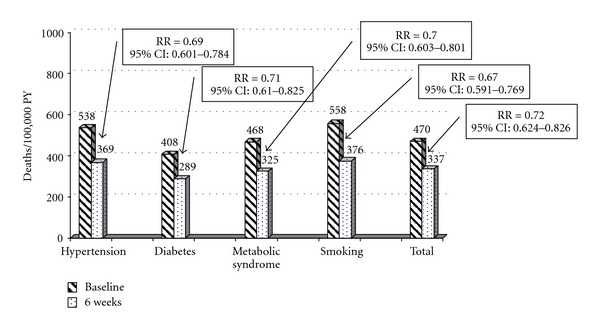
CHD Mortality (Estimated Deaths/100,000 PY). PY = person-years.

**Figure 2:**
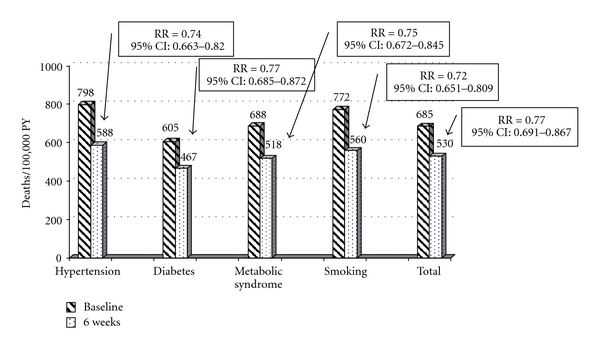
CVD Mortality (Estimated Deaths/100,000 PY). PY = person-years.

The estimates of 10-year CHD mortality based on the SCORE risk at the baseline and six-week assessments are summarized in Figure 1. The number of estimated CHD deaths per 100,000 person-years decreased from 470 to 337 after combination treatment of ezetimibe with the current statin for six weeks. This is equivalent to a 28% reduction in the overall estimated risk for fatal CHD (RR = 0.72; 95% CI: 0.624 to 0.826). Similar decreases in the estimated 10-year CHD mortality were observed for patients with hypertension (RR = 0.69; 95% CI: 0.601 to 0.784), diabetes (RR = 0.71; 95% CI: 0.610 to 0.825), the metabolic syndrome (RR = 0.70; 95% CI: 0.603 to 0.801), and for smokers (RR = 0.67; 95% CI: 0.591 to 0.769). The estimated effect of treatment on total CVD mortality is summarized in Figure 2. These results indicate that, in this cohort of patients, the estimated number of CVD deaths decreased from 685 to 530 per 100,000 person-years for an estimated relative risk of 0.77 (95% CI: 0.689 to 0.867). Similar significant risk reductions were observed for the patient subgroups analyzed.

A low incidence (5.6%) of predominantly mild adverse events with probable or definite causal association to ezetimibe was observed in the Ezetrol Add-On study. The most frequently reported adverse events were constipation (0.7%), diarrhea (0.4%), dizziness (0.4%), flatulence (0.3%), myalgia (0.3%), headache (0.3%), dyspepsia (0.2%), nausea (0.2%), fatigue (0.2%), and arthralgia (0.2%). There were no serious adverse events reported in the study. 

## 4. Discussion

An important proportion of patients with hypercholesterolaemia do not achieve target cholesterol levels with statin monotherapy [10–12]. In these patients, combination of ezetimibe with low-dose statin has been shown to be effective in improving the lipid profile, providing an additional 20% to 25% reduction in low density lipoprotein cholesterol (LDL-C) compared to statin monotherapy [9, 25], while potentially protecting from the risk of adverse events associated with high-dose statins through moderating the dose of co-administrated statin [20–22]. To date, the direct effect of ezetimibe co-administered with a statin on the risk of cardiovascular mortality from a population perspective has not been reported. The current study applied the SCORE model to estimate the change in 10-year risk for cardiovascular mortality in patients that had persistent elevated LDL-C while on statin monotherapy, upon co-administration of ezetimibe with their existing statin regimen. The SCORE cohort consists of 205,178 patients with more than 2.7 million person-years of followup and a total of 7,934 observed cardiovascular deaths (5,652 from coronary heart disease) [7]. The sample size, high number of events, and duration of followup of the cohort ensure high predictive validity of the model. 

The results of the current study indicate that co-administration of ezetimibe 10 mg/day to any statin regimen is effective in reducing the estimated 10-year CHD and CVD mortalities by 25% and 20%, respectively, based on the SCORE risk model. The calculation of mortality risk with the SCORE model is based on changes in the lipid profile as well as changes in SBP and smoking status. Although a change of smoking status is unlikely to occur within six weeks, it cannot be excluded. However, its impact on cardiovascular risk within six weeks is likely limited. Conversely, the change in SBP may have affected the overall risk estimate. In the current study the mean change in SBP between baseline and six weeks of followup was −3.75 mmHg. In fact the change in SBP observed for the total study sample would produce a 6.5% change in estimated CHD risk (RR = 0.935), whereas the observed change in total cholesterol would produce a 24.9% change in the estimated CHD risk (RR = 0.75). These results are in agreement with recent studies showing that co-administration of ezetimibe with statins provides an additional improvement in serum LDL-C and overall lipid profile in patients with inadequate lipid control while on statin monotherapy [14–19]. Furthermore, recent reports indicate that ezetimibe exerts pleiotropic effects on cardiovascular function which are similar [26] or improved [27, 28] compared to statin monotherapy, while additional trials are ongoing which will further analyze the impact of ezetimibe on these effects [29, 30]. 

A number of features in our study design render its results relevant to the real-life setting and generalizable to the target population; patients were recruited without any limitations in their statin regimen and continued receiving it in combination with ezetimibe during the six-week study follow-up period. Furthermore, physicians were instructed to adhere to their routine care for the management of their patients, and to the product monograph for ezetimibe treatment. Finally, generalization of our study results to the real-life setting is further supported because the inclusion and exclusion criteria of the current study were defined in order to represent the patients treated with ezetimibe in routine clinical practice and because all patients were included in the analysis, regardless of compliance with treatment. As a result, the demographic profile and distribution of statins used by the subjects in our study were similar to those reported in population-based epidemiological Canadian studies [10, 31]. 

Potential limitations of the current study are related to the single cohort design. However, this was an observational study with primary objective, the change in estimated 10-year CVD mortality risk during ezetimibe add-on to the existing statin regimen, and not the comparison of its effectiveness to a different treatment. Furthermore, taking into consideration that hypercholesterolaemia in all patients was inadequately controlled while on a steady and established statin regimen, it is unlikely that maintenance on the current statin regimen would incur any further lipid changes, which serves as an internal control. Another potential limitation of the current study may involve the use of the SCORE model in a North American population. The SCORE model was developed in Europe and may reflect specific risk factor effects of the European population. However, the SCORE model was selected for the current study because it specifically produces estimates of mortality and not CVD in general. In addition, the wide range of European-based ethnic origins in the Canadian population renders the use of the SCORE model even more relevant. Finally, the generalizability of our results to the target population and the emulation of the real-life setting achieved by the study design are important strengths of the current study, while the use of a validated model as the outcome measure allows reproducibility and comparisons across different studies and populations.

## 5. Conclusions

For patients who remain at increased risk for cardiovascular morbidity or mortality due to persistent hypercholesterolaemia while on statin monotherapy, co-administration of ezetimibe with a statin is effective in significantly reducing the estimated long-term risk for cardiovascular mortality. However, additional population studies to confirm the long-term effect of combination therapy with ezetimibe and statins on cardiovascular mortality are required. 

##  Conflict of Interest

JSS Medical research was hired by Merck & Co to conduct this study and perform the data analysis. S. Boukas and J. S. Sampalis are employees of JSS Medical Research Inc.; S. Bissonnette was an employee of Merck & Co during the analysis and writing the paper. This study was supported by Merck & Co and JSS Medical Research Inc.

## Figures and Tables

**Table 1:** Modifiable SCORE risk factors at baseline and final (6-week) assessments.

	Systolic Blood Pressure (mmHg)				Total Cholesterol (mmol/L)			
Comorbidity/Risk Factor*	Baseline	6-week	Absolute Change		Baseline	6-week	Absolute Change	
	Mean (SD)	Mean (SD)	Mean (SD)	95% CI	Mean (SD)	Mean (SD)	Mean (SD)	95% CI

All Patients (*N* = 825)	132.0 (14.0)	128.2 (13.7)	−3.75 (13.3)	−2.84, −4.66	5.60 (1.00)	4.40 (1.00)	−1.18 (0.89)	−1.12, −1.24
Hypertension (*N* = 423)	138.6 (13.3)	132.0 (12.9)	−6.59 (14.1)	−5.25, −7.94	5.51 (0.92)	4.36 (0.98)	−1.14 (0.91)	−1.05, −1.23
Diabetes (*N* = 342)	132.7 (13.7)	129.2 (13.9)	−3.49 (13.5)	−2.06, −4.93	5.63 (1.11)	4.51 (1.08)	−1.26 (0.94)	−1.16, −1.36
Metabolic Syndrome (*N* = 395)	136.0 (13.9)	131.7 (13.0)	−4.27 (13.9)	−2.89, −5.64	5.62 (0.99)	4.35 (1.04)	−1.27 (0.97)	−1.17, −1.37
Current Smokers (*N* = 188)	132.1 (14.4)	126.6 (13.6)	−5.50 (13.1)	−3.62, −7.38	5.67 (1.18)	4.34 (0.92)	−1.33 (0.97)	−1.47, −1.19

*Patients may have more than one comorbid condition.

**Table 2:** CHD SCORE at baseline and final (6-week) assessments.

Patient Group	CHD SCORE															
	Baseline				6 weeks				Absolute Change				Percent Change			

	Mean	SD	95% CI		Mean	SD	95% CI		Mean	SD	95% CI		Mean	SD	95% CI	
			Lower	Upper			Lower	Upper			Lower	Upper			Lower	Upper

Hypertension	0.054	0.044	0.050	0.058	0.037	0.030	0.034	0.040	−0.017	0.023	−0.019	−0.015	−0.280	0.243	−0.304	−0.257
Diabetes	0.041	0.034	0.037	0.044	0.029	0.025	0.026	0.032	−0.012	0.017	−0.014	−0.010	−0.264	0.244	−0.290	−0.238
MS*	0.047	0.040	0.043	0.051	0.033	0.028	0.030	0.035	−0.014	0.020	−0.016	−0.012	−0.273	0.250	−0.298	−0.248
Smoking	0.056	0.050	0.049	0.063	0.038	0.034	0.033	0.042	−0.018	0.023	−0.021	−0.014	−0.303	0.211	−0.334	−0.273
Total	0.047	0.040	0.044	0.050	0.034	0.029	0.032	0.036	−0.013	0.020	−0.015	−0.012	−0.254	0.243	−0.271	−0.238

*MS: metabolic syndrome.

**Table 3:** Total CVD SCORE at baseline and final (6-week) assessments.

Patient Group	Total CVD SCORE															
	Baseline				6 weeks				Absolute Change				Percent Change			

	Mean	SD	95% CI		Mean	SD	95% CI		Mean	SD	95% CI		Mean	SD	95% CI	
Lower			Upper			Lower	Upper			Lower	Upper			Lower	Upper

Hypertension	0.080	0.064	0.074	0.086	0.059	0.048	0.054	0.063	−0.021	0.032	−0.024	−0.018	−0.230	0.243	−0.253	−0.207
Diabetes	0.061	0.051	0.055	0.066	0.047	0.041	0.042	0.051	−0.014	0.024	−0.017	−0.012	−0.200	0.255	−0.227	−0.173
MS*	0.069	0.059	0.063	0.075	0.052	0.045	0.047	0.056	−0.017	0.028	−0.020	−0.014	−0.212	0.257	−0.238	−0.187
Smoking	0.077	0.070	0.067	0.087	0.056	0.051	0.049	0.063	−0.021	0.030	−0.025	−0.016	−0.254	0.214	−0.284	−0.223
Total	0.068	0.059	0.065	0.072	0.053	0.046	0.050	0.056	−0.015	0.028	−0.017	−0.013	−0.198	0.249	−0.215	−0.181

*MS: metabolic syndrome.
